# Perforated Appendiceal Mucinous Neoplasm With Long-Term Progression to Pseudomyxoma Peritonei in an Elderly Patient: A Case Report

**DOI:** 10.7759/cureus.96973

**Published:** 2025-11-16

**Authors:** Mohamed Saifaldin Eltayieb Mohamed, Doaa Mahmoud Akasha Mohamed, Mahmood Dardas, Mustafa Kraidi, Waleed Afifi

**Affiliations:** 1 Acute Medicine, Surrey and Sussex Healthcare NHS Trust, Redhill, GBR; 2 Pediatrics, University of Gezira, Wad Madani, SDN; 3 Acute Medicine, East Surrey Hospital, Surrey and Sussex Healthcare NHS Trust, Redhill, GBR

**Keywords:** appendiceal mucinous neoplasm, appendiceal mucocele, lamn, low-grade appendiceal mucinous neoplasms, low-grade mucinous appendiceal neoplasm, mucinous ascites, mucinous neoplasm of appendix, omental nodularity, perforated appendix, pseudomyxoma peritonei

## Abstract

Low-grade appendiceal mucinous neoplasms (LAMNs) are rare tumors that may present as appendiceal mucoceles, with perforation carrying the risk of mucin dissemination and subsequent pseudomyxoma peritonei (PMP). We report the case of an 83-year-old female who presented with lower abdominal pain and was found on CT imaging to have an appendiceal mucocele. Laparoscopic appendicectomy revealed a perforated appendiceal mucocele with disseminated mucin, and histopathology confirmed a pT4a LAMN with perforation at the tip and a clear base. Despite its initially indolent course, surveillance over two and a half years demonstrated progressive intra-abdominal mucin accumulation and omental nodularity, culminating in radiological features of PMP with large-volume ascites and enhancing septations. Given her age and comorbidities, the patient declined cytoreductive surgery and was managed palliatively. This case emphasizes the indolent but progressive nature of perforated LAMNs, the importance of long-term surveillance, and the need to tailor management according to patient preference and surgical risk, particularly in elderly individuals. Clinicians should maintain a high index of suspicion for malignancy in appendiceal mucoceles and ensure ongoing surveillance following perforated LAMN to detect and manage progression to PMP.

## Introduction

Appendiceal mucinous neoplasms (AMNs) are rare, accounting for less than 1% of appendiceal pathologies and approximately 0.2%-0.7% of all appendectomies [1, 2]. Low-grade appendiceal mucinous neoplasms (LAMNs) are typically indolent but may rupture, resulting in mucin dissemination and subsequent pseudomyxoma peritonei (PMP), a condition characterized by the progressive accumulation of gelatinous ascites and mucinous implants on peritoneal surfaces [3, 4]. Standard management often includes cytoreductive surgery with hyperthermic intraperitoneal chemotherapy (HIPEC); however, treatment decisions may be challenging in elderly patients, those unfit for aggressive interventions, or those who refuse invasive procedures [5, 6].

While previous reports have described the clinical and surgical features of LAMNs, few studies provide detailed long-term radiological and biochemical follow-up in elderly patients who decline surgery. The objective of this case report is to document the long-term radiological progression of a perforated LAMN to PMP in an elderly patient who declined cytoreductive surgery, highlighting the natural disease course and the importance of individualized, surveillance-based management [7-15]. By presenting this case, we aim to emphasize the importance of vigilant long-term monitoring, the potential utility and limitations of tumor markers, and the role of tailored management strategies in high-risk or surgically unfit patients.

## Case presentation

An 83-year-old female with a history of hypertension and hypercholesterolemia presented to the ED with a 3-day history of worsening lower abdominal pain, initially accompanied by loose stools. The pain was constant and exacerbated by movement and coughing. On examination, there was clinical suspicion of acute appendicitis, and a contrast-enhanced CT of the abdomen and pelvis was performed.

CT abdomen and pelvis findings

**Figure 1:**
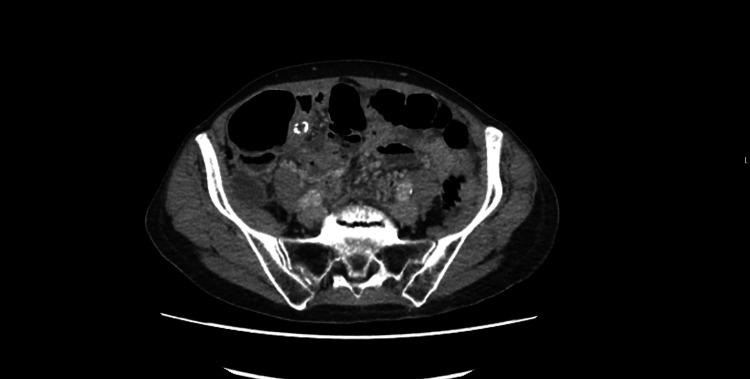
Axial contrast-enhanced CT of the abdomen and pelvis demonstrating a fluid-filled, dilated tubular structure in the right lower abdomen with mural calcification at its base, continuous with the cecum, consistent with a possible appendiceal mucocele.

**Figure 2:**
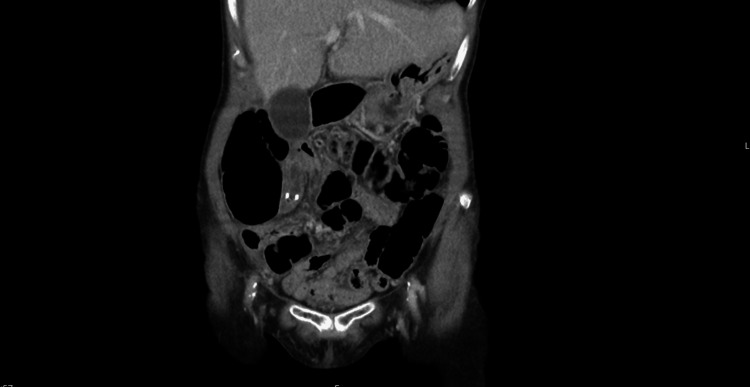
Coronal CT of the abdomen and pelvis with intravenous contrast demonstrating the same fluid-filled appendiceal lesion with mural calcification, in continuity with the cecum.

Imaging demonstrated an ill-defined, fluid-filled tubular structure in the right lower abdomen. It measured approximately 8.5 mm in thickness with peripheral calcification at its base and appeared to be continuous with the caecum (Figures 1-2). The working diagnosis was a possible mucocele of the appendix.

Laparoscopic appendicectomy was performed for suspected perforated appendiceal mucocele versus acute appendicitis.

Intraoperative findings

A bulky, swollen, and inflamed appendix, perforated at the tip and adherent to the small bowel loops and caecum, was identified, with the appendiceal base appearing healthy. During the operation, copious disseminated mucin was noted throughout the peritoneal cavity, including between bowel loops, in the pelvis, and in the perihepatic region, where an appendiceal mucinous tumour could not be excluded. No formal surgical excision or peritonectomy was performed, and although 5.5 L of sterile water was used for extensive peritoneal washout, complete removal of the mucin was not achieved or documented in the operation note.

Postoperative course

The patient developed a postoperative ileus on day three, which was managed conservatively with a nasogastric tube and potassium replacement. Due to high drain output, poor clinical progress, and an inability to tolerate oral intake, a repeat CT abdomen and pelvis was performed on day 8, which confirmed a small bowel obstruction. This resolved spontaneously after 10 days, although abdominal distension persisted. The patient was discharged on day 15 following drain removal and had recovered satisfactorily.

Four days after discharge, she was readmitted with dizziness and malaise. A repeat CT abdomen and pelvis showed no new abnormalities, and she was discharged the same day.

Investigations and follow-up

Histopathology confirmed a pT4a LAMN, perforated at the tip with mucin present on the serosal surface. The appendiceal base was free of tumour. Apart from copious disseminated mucin, there was no evidence of peritoneal deposits at that time. The histopathology sample was obtained during the laparoscopic appendicectomy.

Staging and surveillance

A staging CT chest with contrast showed no evidence of metastasis, and a colonoscopy performed 8 weeks later demonstrated only diverticulosis. Serial tumour markers were monitored as part of ongoing surveillance (Table 1). The decision to perform full staging was made during a multidisciplinary team (MDT) discussion, with additional input from a tertiary center specializing in PMP. The final consensus was for active radiological surveillance with regular CT scans, an approach deemed appropriate given the low-grade nature of the tumour, negative baseline imaging, and the need to balance the risk of progression against the morbidity of immediate cytoreductive surgery. The patient’s preference to avoid invasive surgical intervention was also incorporated into the management plan.

**Table 1:** A summary of the tumour marker trends. CEA: Carcinoembryonic Antigen; CA 19-9: Carbohydrate Antigen 19-9; CA 125: Cancer Antigen 125.

Tumour Marker	Value (1 month from initial presentation)	Value (around 10 months later)	Reference Range	Trend / Interpretation
CEA	<1.7	3.9	0-4.9 µg/L	Remained within normal range
CA 19-9	-	3	0-36 U/mL	Within normal range
CA 125	37	91	0-34 U/mL	Progressive rise reflecting CT evidence of mild disease progression; no intervention undertaken as the patient declined surgery

At approximately 11 months, follow-up CT demonstrated mild disease progression, with persistent perihepatic fluid and newly prominent paracardiac lymph nodes. A concurrent rise in CA 125 levels was observed (Table 1), corresponding with early radiological evidence of progression. However, no immediate intervention was undertaken, as the patient declined surgical management and preferred continued active surveillance.

**Figure 3:**
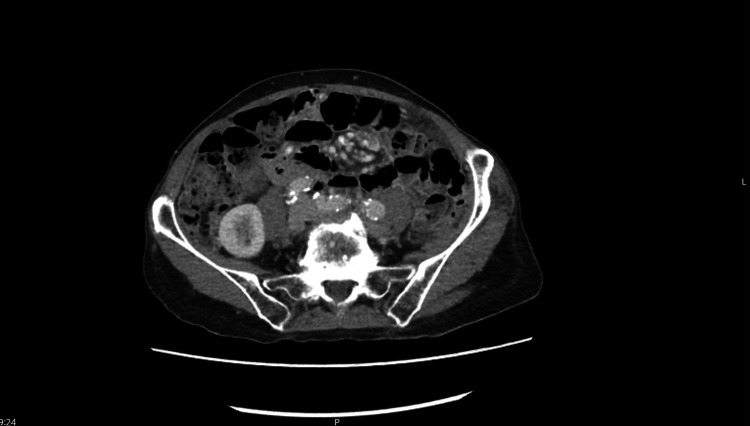
CT abdomen and pelvis with IV contrast showing mildly enlarged anterior paracardiac lymph nodes, progressive ascites, and multiple newly developed left-sided omental nodules, consistent with evolving peritoneal disease.

**Figure 4:**
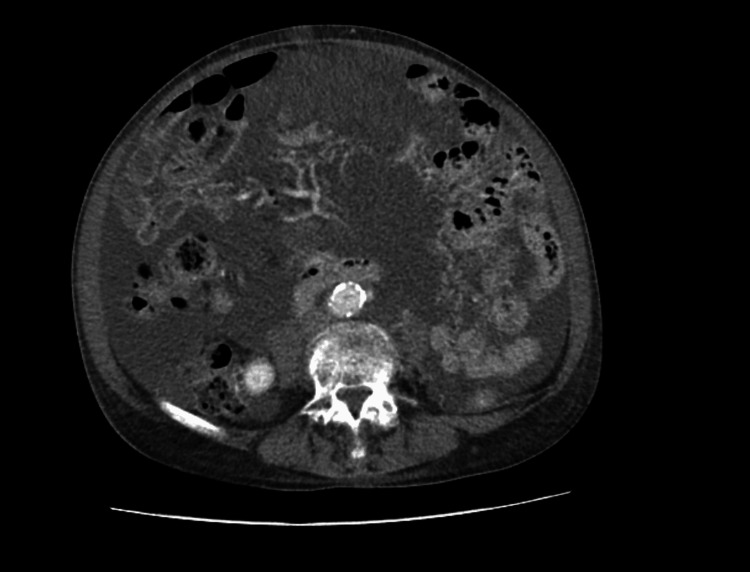
CT abdomen and pelvis with IV contrast showing omental nodularity, cardiophrenic lymphadenopathy, and diffuse intra-abdominal free fluid, consistent with peritoneal disease progression.

At 18 months, repeat CT showed further disease progression with increased intra-abdominal free fluid and newly developed multiple omental nodules, predominantly on the left side (Figure 3). Approximately one year later, the patient was reviewed in the ambulatory care clinic, where a repeat CT abdomen and pelvis demonstrated omental nodularity and cardiophrenic lymph nodes, with diffuse intra-abdominal free fluid consistent with peritoneal disease progression (Figure 4). An ultrasound of the abdomen performed by interventional radiology for potential drainage revealed an abnormality in the lower abdomen, appearing as a multicystic mass with numerous septations and loculi, rendering it unsuitable for percutaneous drainage and highly suggestive of PMP. The drainage procedure was therefore cancelled. As the patient declined major surgery, a decision was made to continue with a palliative approach to preserve quality of life.

## Discussion

Diagnostic considerations

This case details the complex clinical course of an 83-year-old woman who presented with a perforated appendiceal mucocele, later confirmed as a pT4a LAMN, ultimately progressing to pseudomyxoma peritonei (PMP). The diagnosis of LAMN can be challenging, as symptoms and imaging findings often mimic acute appendicitis [8, 9]. Certain radiological features, such as a fluid-filled, dilated appendix with mural or peripheral calcification, should raise suspicion for a mucinous neoplasm rather than simple inflammation [10]. Preoperative recognition is critical, as intraoperative rupture can result in mucin spillage and subsequent PMP development [1-4, 11]. A mucocele represents a dilated, mucus-filled appendix, which may be benign or neoplastic [2]. Distinguishing between acute appendicitis and a perforated mucinous neoplasm preoperatively is therefore essential for appropriate surgical planning [1, 7].

Surgical management

In this case, laparoscopic appendicectomy was initially undertaken for suspected appendicitis, but intraoperatively a perforated mucocele with disseminated mucin was identified. Once spillage occurs, as in pT4a disease, the risk of developing PMP increases substantially [3, 4, 11]. The gold-standard surgical approach for suspected LAMN is en-bloc resection with intact specimen retrieval to prevent peritoneal seeding [3]. When perforation is discovered, referral to a peritoneal malignancy centre is recommended to assess candidacy for cytoreductive surgery (CRS) with hyperthermic intraperitoneal chemotherapy (HIPEC) [5]. In elderly or comorbid patients, CRS/HIPEC may carry prohibitive risks, necessitating a carefully balanced, patient-centred approach that considers operative morbidity alongside patient preferences [6, 14].

Surveillance and progression

This case illustrates the indolent but progressive nature of perforated LAMN. Radiological evolution was documented over long-term surveillance, progressing from minimal peritoneal fluid to established PMP with omental nodularity, enhancing septations, and multicystic, septated ascites, classic radiological hallmarks of PMP [12, 13, 15]. Despite the absence of early peritoneal deposits, disease progression became evident on interval CT scans. These findings underscore the need for structured, long-term follow-up, including serial imaging and tumour marker monitoring, even in patients who remain clinically stable [6, 13]. Structured surveillance programs for LAMNs have been shown to improve early detection of PMP and guide timely management [7, 15].

Tumor markers and limitations

Tumor markers such as CEA, CA 19-9, and CA 125 are commonly used in LAMN and PMP surveillance. In this patient, CA 125 demonstrated a progressive rise corresponding with mild disease progression on imaging, while CEA and CA 19-9 remained within normal limits [6, 12]. Rising CA 125 may reflect peritoneal irritation or increasing mucin burden; however, these markers lack sensitivity and specificity for predicting PMP progression and should be interpreted alongside imaging findings. Their primary utility lies in trend monitoring rather than definitive diagnosis or prognosis [6, 12].

Therapeutic decision-making

For established PMP, CRS with HIPEC remains the only potentially curative option [5]. However, given the patient’s advanced age, comorbidities, and preference to avoid major surgery, management prioritized palliation and quality of life. Ascitic drainage was not attempted due to the multicystic and septated nature of the fluid, typical of PMP, which limits the effectiveness of simple paracentesis [2, 12]. This case highlights the importance of shared decision-making, respecting patient autonomy, and tailoring management to balance the potential benefit of curative intervention against operative risk [14]. When surgery is declined or contraindicated, supportive care, including symptom control and periodic imaging, forms the cornerstone of management [7, 15].

## Conclusions

This case of perforated LAMN progressing to PMP provides key clinical insights that reinforce and expand the current understanding of AMNs. The presence of an appendiceal mucocele, particularly when associated with mural calcification or perforation, should immediately raise suspicion for malignancy, as delayed or incomplete recognition can lead to mucin spillage and subsequent PMP. Surgical management must prioritise en-bloc resection with an intact specimen to prevent rupture, and once perforation (pT4a) has occurred, early referral to a specialised peritoneal malignancy centre for evaluation for CRS and HIPEC is essential.

All patients with perforated LAMN should undergo long-term, structured surveillance with serial CT imaging and tumour marker monitoring. In this case, a progressive rise in CA 125 correlated with subtle radiological progression, illustrating both the potential utility and the limitations of tumour markers in predicting disease evolution. For elderly patients or those with significant comorbidities who decline or are unsuitable for major surgery, management should focus on maintaining quality of life through palliative surveillance and multidisciplinary care.

By documenting the gradual radiological and biochemical progression of perforated LAMN in an elderly patient who declined surgical intervention, this report contributes to the growing body of literature describing the natural history, diagnostic challenges, and long-term outcomes of conservatively managed cases. It highlights the importance of early recognition, vigilant follow-up, and patient-centred decision-making in optimising care for this rare but clinically significant condition.
